# Editorial Profile of the BJCVS’ Present, Past, and Future

**DOI:** 10.21470/1678-9741-2024-0997

**Published:** 2024-11-18

**Authors:** Marcos Aurélio Barboza de Oliveira, Andréia Cristina Feitosa do Carmo, Camila Sáfadi Alves Gonçalves, Paulo Roberto Barbosa Évora

**Affiliations:** 1 Department of Pediatric Cardiovascular Surgery, Hospital de Messejana, Fortaleza, Ceará, Brazil; 2 Hospital São Paulo, Escola Paulista de Medicina (EPM), Universidade Federal de São Paulo (UNIFESP), São Paulo, São Paulo, Brazil; 3 Sociedade Brasileira de Cirurgia Cardiovascular, São Paulo, São Paulo, Brazil; 4 Faculdade de Medicina de Ribeirão Preto (FMRP), Universidade de São Paulo (USP), Ribeirão Preto, São Paulo, Brazil

The Brazilian Journal of Cardiovascular Surgery (BJCVS) stands as the premier publication dedicated to cardiovascular surgery and related themes throughout Latin America. Continuously published since 1986, it remains an essential resource, receiving contributions daily from across the globe.

Under the leadership of Prof. Dr. Paulo Roberto Barbosa Evora, as Editor-in-Chief, and Prof. Dr. Walter José Gomes, as Co-Editor, alongside 26 esteemed Associate Editors and a global network of reviewers, BJCVS is committed to disseminating cutting-edge research in our field.

**Fig. 1:**
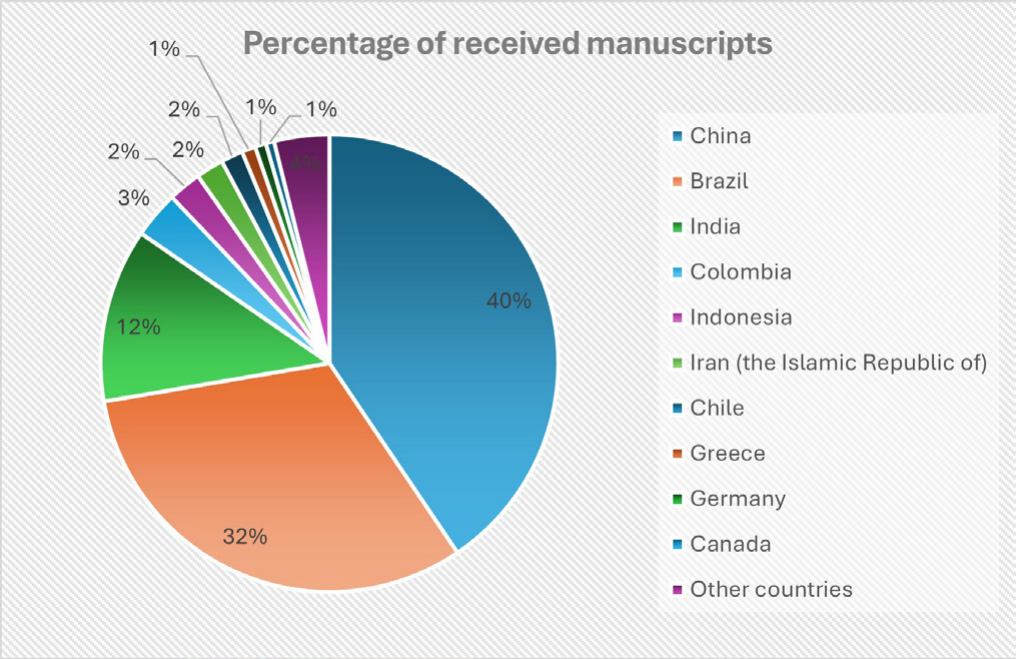
Number of manuscripts received by country of submission since 01.01.2023.

Since early 2023, we have received remarkable 874 submissions (Table 1) from 50 different countries (the top 10 are displayed in Figure 1), with almost 40 new articles per month undergoing rigorous peer review by our skilled reviewers. Their dedication to advancing scientific discourse is invaluable, and we owe a debt of gratitude to each of them.

**Table 1:** Number of articles received since 01.01.2023.

Manuscript type	Original	Percentage of total
Original Article	570	65.2%
Review Article	77	8.8%
How to do it	67	7.7%
Letters to the Editor	52	5.9%
Case Report	38	4.3%
Brief Communication	33	3.8%
Multimedia	16	1.8%
Special Article	15	1.7%
Editorial	5	0.6%
Guidelines	1	0.1%
Total	874	100.0%

In the last year, according to data from wisdom.ai®, BJCVS welcomed 553 new authors and 189 returning ones from over 39 countries, resulting in the publication of 142 articles. Notably, these articles garnered 1,158 citations in high-impact journals worldwide, with significant attention from regions such as China, India, Turkey, and Latin America^[1]^.

**Fig. 2:**
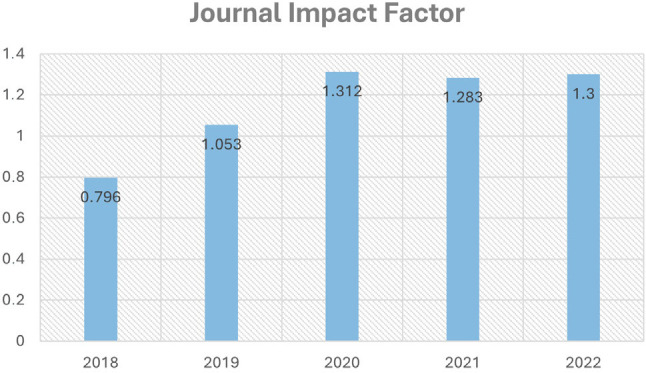
Evolution of Journal Impact Factor of the Brazilian Journal of Cardiovascular Surgery (BJCVS) from 2018 to 2022.

Our Journal Citation Reports (JCR) metrics reflect this upward trajectory, showcasing the growing influence and relevance of BJCVS within the scientific community. The Journal Impact Factor has seen a stable increase from 0.796 in 2018 to 1.3 in 2022, demonstrating our journal's enhanced visibility and citation in the field (Figure 2), not to mention it is ranked in top 10 Journals in CTSNet site (https://www.ctsnet.org/journals) with higher JCR scores. These metrics are derived from the 2022 data compiled by the Web of Science Core Collection, the leading collection of quality journals, books, and conference proceedings in the world’s largest publisher-neutral global citation database. Publications are evaluated by a global team of in-house editors at Clarivate using rigorous selection criteria. The data from selected content are then carefully curated to ensure accuracy in the JCR metrics, together with a wide body of descriptive data. These insights enable researchers, publishers, editors, librarians, and funders to explore the key drivers of a journal’s value for diverse audiences, affirming the BJCVS' commitment to publishing high-quality research and its recognition within the global scientific community^[2,3]^.

Our bulletin is featuring new team members tasked with conducting interviews, incorporating multimedia elements, reporting breaking news, and including cultural materials. These additions aim to expand the scope of cardiovascular themes covered and to establish a novel interface with leading specialists globally.

The trend of scientific publications in the form of "short" texts has gained prominence recently. These texts are concise and straightforward summaries of full-length scientific papers, allowing readers to gain a quick and clear overview of key research points and findings. In the context of the BJCVS, the use of "short" texts can be a powerful strategy to increase the reach and accessibility of published research.

Implementing "short" texts can significantly increase the visibility and impact of research published in the journal, in addition to facilitating the dissemination of knowledge among professionals and those interested in the field of cardiovascular surgery.

Together, we will continue to advance the frontiers of cardiovascular surgery research and foster collaboration across borders for the betterment of patient care worldwide.
